# Out-of-plane surface patterning by subsurface processing of polymer substrates with focused ion beams

**DOI:** 10.3762/bjnano.11.151

**Published:** 2020-11-06

**Authors:** Serguei Chiriaev, Luciana Tavares, Vadzim Adashkevich, Arkadiusz J Goszczak, Horst-Günter Rubahn

**Affiliations:** 1 NanoSYD, Mads Clausen Institute, University of Southern Denmark, Alsion 2, Sønderborg, 6400, Denmark; 2 Centre for Industrial Electronics, Department of mechanical and electronic engineering, University of Southern Denmark, Alsion 2, Sønderborg, 6400, Denmark

**Keywords:** direct patterning, focused helium ion beam, out-of-plane nanopatterning, polymers, thin films

## Abstract

This work explores a new technique for the out-of-plane patterning of metal thin films prefabricated on the surface of a polymer substrate. This technique is based on an ion-beam-induced material modification in the bulk of the polymer. Effects of subsurface and surface processes on the surface morphology have been studied for three polymer materials: poly(methyl methacrylate), polycarbonate, and polydimethylsiloxane, by using focused ion beam irradiation with He^+^, Ne^+^, and Ga^+^. Thin films of a Pt_60_Pd_40_ alloy and of pristine Au were used to compare the patterning of thin films with different microstructures. We show that the height of Pt_60_Pd_40_ thin films deposited onto poly(methyl methacrylate) and polycarbonate substrates can be patterned by He^+^ ion beams with ultrahigh precision (nanometers) while preserving in-plane features, at the nanoscale, of the pre-deposited films. Ion irradiation of the Au-coated samples results in delamination, bulging, and perforation of the Au film, which is attributed to the accumulation of gases from radiolysis at the film–substrate interface. The irradiation with Ne^+^ and Ga^+^ ions destroys the films and roughens the surface due to dominating sputtering processes. A very different behavior, resulting in the formation of complex, multiscale 3D patterns, is observed for polydimethylsiloxane samples. The roles of the metal film structure, elastic properties of the polymer substrate, and irradiation-induced mechanical strain in the patterning process are elaborated and discussed.

## Introduction

Micro- and nanofabrication with focused ion beams (FIBs) is currently a subject of strong interest within diverse fields of materials science and technology [1]. In recent years, the capabilities of FIBs have been substantially enhanced leading to a broad range of applications by the implementation of light ion beams (He^+^ and Ne^+^) emitted by a gas field ion source (GFIS). This has enabled direct, maskless surface patterning with a superior lateral resolution and depth control [2–3]. The portfolio of the currently used FIB-based and FIB-assisted surface patterning techniques includes a number of different methods, such as ion-beam sputtering of surface layers (ion-beam milling), ion-beam-assisted chemical etching and ion-beam-assisted chemical vapor deposition [1–3]. All these methods are based on processes that either add or remove atoms on the surface or in the subsurface atomic layers.

The ion beams deposit their energy and, therefore, affect the structure and properties of materials over the entire depth of their penetration path in a target. In our recent work [4], we demonstrated that, in addition to the direct surface patterning by the abovementioned techniques, the radiation damage generated by He^+^ FIB in the bulk of poly(methyl methacrylate) (PMMA) substrates can be used for well-controlled and nanometer-precise patterning of the height of metal thin films and nanostructures prefabricated on the surface of these substrates. This technique is based on subsurface chemical decomposition, structural reconstruction, and, as a result of these processes, volume shrinkage of the PMMA polymer under ion irradiation [5–7]. The most important physical and chemical phenomena behind this material modification include scission and cross-linking of polymer chains, which can occur simultaneously, as well as the formation of volatile molecules and their desorption from the polymer bulk [7]. In fact, the method utilizes ion energy losses to manipulate the surface morphology by means of radiation damage generated in the substrate bulk and minimizes the surface damage resulting from sputtering. This leaves the thin films and the prefabricated thin-film nanostructures on the PMMA surface essentially intact and provides a new route to their out-of-plane patterning, which is interesting for a range of thin film applications.

In the current work, we extend our study to the effects of the ion mass by irradiating PMMA substrates with He^+^, Ne^+^, and Ga^+^ ions, and to the role of pathways for volatile radiolysis products to leave the irradiated material. We also investigate the possibility to pattern the surface of other polymer substrates, such as polycarbonate (PC) and polydimethylsiloxane (PDMS), by subsurface processing with He^+^ ions. The choice of materials for this work has been directed by their diverse applications in micro- and nanotechnology and by the high susceptibility of their structure to ion irradiation [5]. Another important aspect is that the chosen materials are different in their chemical structure, chemical composition, and mechanical properties. This is the basis of a comparative study of the role of material-related factors in the FIB-induced surface patterning. PMMA and PC polymers are especially interesting for many reasons: PMMA is widely used as a positive resist for X-ray, deep UV [8], electron and ion-beam lithography [9]. Structural transformation and volume shrinkage of PMMA under ion irradiation have been reported in several publications [6–7,9–13]. PC is the second most sold thermoplastic polymer. It is extensively used in microtechnology due to its excellent optical, mechanical, and chemical properties [14]. Compared to PMMA, it has a much higher mechanical toughness, thermal resistance, chemical stability, and as PMMA, it is widely used in optical applications. A range of publications show that, owing to its radiation susceptibility, PC can be used as a positive or negative resist for electron beam lithography [15–16]. It has also been demonstrated that it acts as a type of ion-beam resist in the fabrication of micro- and nanopore membranes and templates for nanowires by chemical etching of through-holes along ion tracks produced by high-energy ions [17–18].

In contrast to PMMA and PC polymers, PDMS is a mineral-organic polymer (its structure includes both carbon and silicon atoms). It is an elastomer and its elasticity can be tuned within a very broad range by changing the degree and the type of polymerization and by post-curing treatments [19–20]. The high and easily tunable elasticity, combined with high transparency, biocompatibility, and low cost, enable the broad use of PDMS for the fabrication of microfluidic, microelectromechanical, and microoptical devices [20]. The effects of ion irradiation on chemical and physical properties and on the surface morphology of PDMS have been extensively investigated [21–24]. It has been shown that the ion beam irradiation can result in a significant compacting and, under certain conditions, in swelling of the irradiated PDMS areas [25]. In addition, a stiff “skin” layer produced by ion irradiation on the PDMS surface leads to the formation of ordered wrinkle-like micropatterns [23–24].

In this work, we have employed thin films of a Pt_60_Pd_40_ alloy and of pristine Au. The primary reason for this choice was the difference in their microstructures, specifically in the availability of structural defects capable of providing the release of gases from radiolysis. As it has been shown before [4], the as-deposited 15 nm Pt_60_Pd_40_ thin films contain arrays of nanoscale cracks. In contrast, our studies have not revealed any cracks or other discontinuities in the as-deposited Au thin films. To study the effects of the ion mass, ultrathin (5 nm thick) Pt_60_Pd_40_ films were used in order to minimize energy losses of Ne^+^ and Ga^+^ ions in these films. In all other cases, such as patterned Pt_60_Pd_40_ films on PC and PDMS substrates and patterned Au films on PMMA substrates, 15 nm metal thin films were used to facilitate the comparison with previously published results [4], in which 15 nm Pt_60_Pd_40_ films were patterned. Also, for the same reason of a more direct comparison, 200 nm thick PMMA substrates were used in this work to study possible effects of changing ion masses.

## Results and Discussion

### Irradiation of PMMA

**Figure 1:**
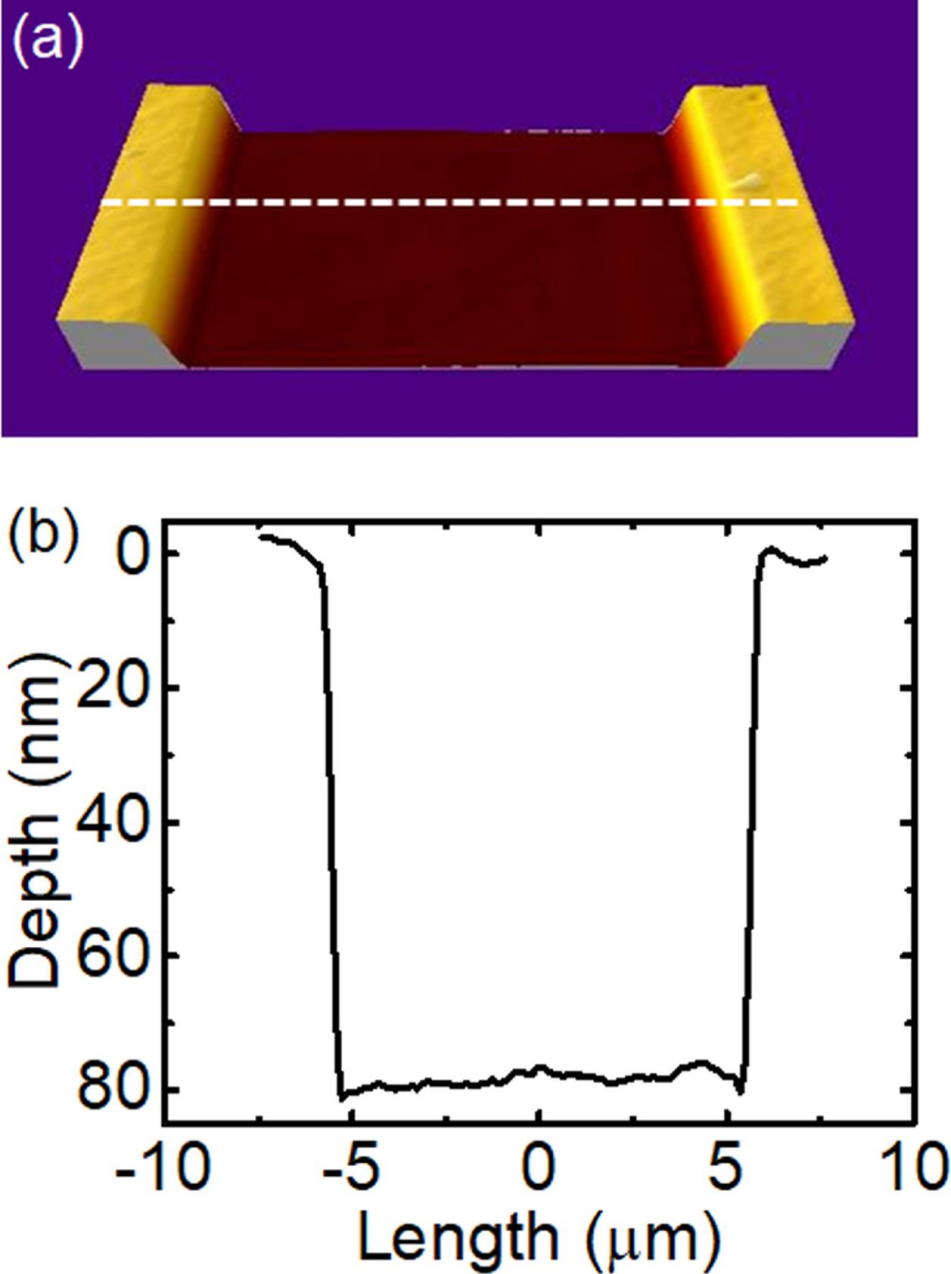
(a) AFM image and (b) the corresponding depth profile of a fragment of the surface depression produced in a 5 nm Pt_60_Pd_40_/200 nm PMMA sample by irradiation with He^+^ FIB at an energy of 25 keV and with a fluence of 1.0 × 10^16^ cm**^−^**^2^. The dashed line in (a) indicates the place of the depth profile.

Figure 1 shows an example of an atomic force microscopy (AFM) image and the corresponding depth profile for a surface region of the Pt_60_Pd_40_/PMMA sample irradiated with He^+^ FIB at a fluence of 1.0 × 10^16^ cm^−2^. It is evident that the irradiation homogeneously lowers the entire irradiated surface to a depth of approx. 80 nm. For convenience, we define the value of the surface depression as a reduction in the surface height (or as a change in the surface depth), for which the baseline values correspond to the non-irradiated area. Patterns of similar shape have been observed for the entire fluence range of the irradiation with He^+^ ions, and also for the irradiation with Ne^+^ and Ga^+^ ions.

**Figure 2:**
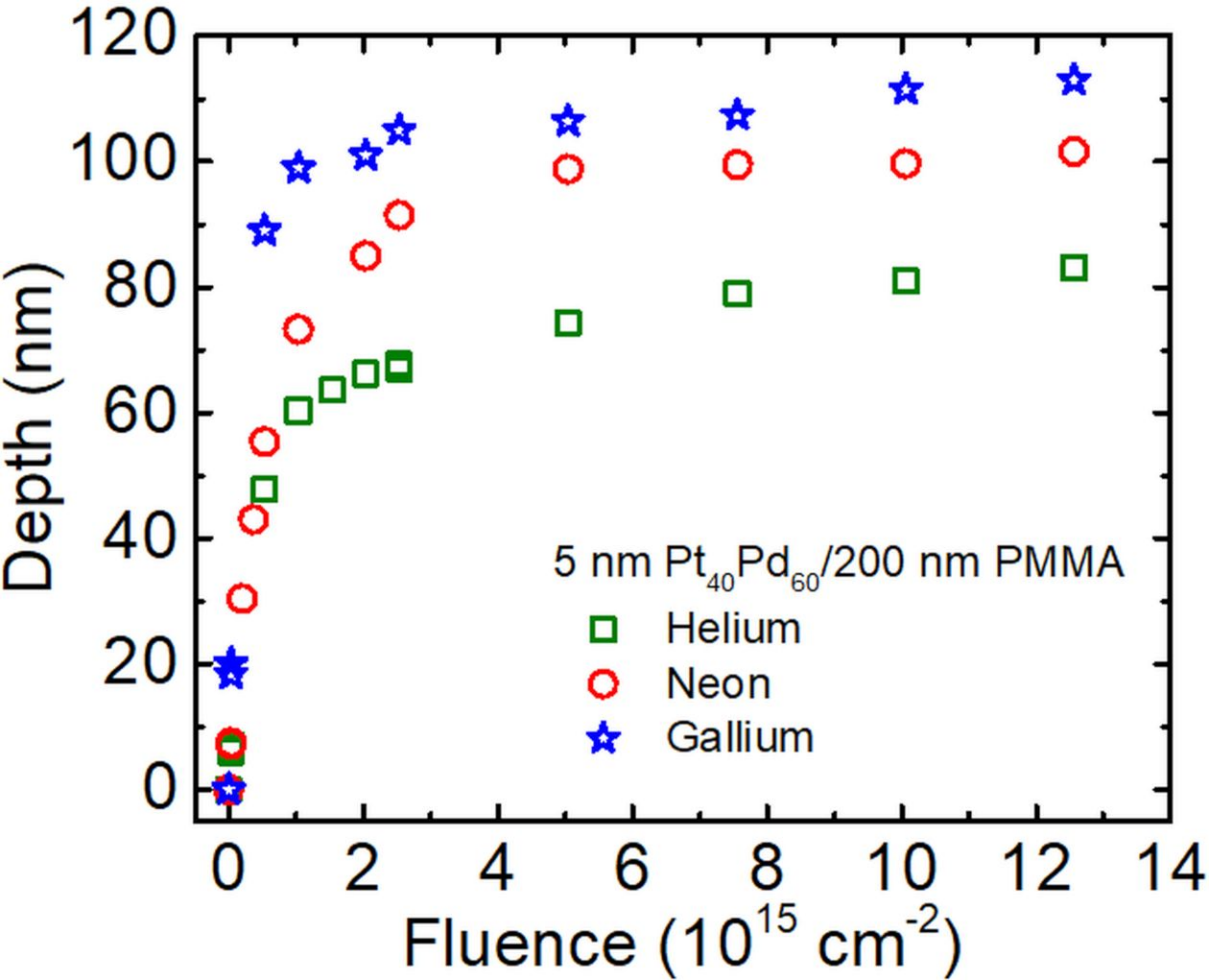
Fluence dependence on the irradiation-induced surface depthening for a 5 nm Pt_60_Pd_40_/200 nm PMMA sample, irradiated with 25 keV He^+^, Ne^+^, and Ga^+^ FIBs.

Figure 2 summarizes the surface depthening as a function of the irradiation fluence for He^+^, Ne^+^, and Ga^+^ ions. All curves demonstrate a very steep increase in depth with increasing fluence in the low-fluence range, followed by a saturation when the fluence increases. The influence of the ion type on the surface depthening is evident from the comparison of these plots: Both the depth-change rate at low-fluence values and the depth saturation level increase with an increase in the ion mass. From a linear regression of the dependence in Figure 2, in the low-fluence range (up to a fluence of 5.0 × 10^14^ cm^−2^), the surface depression rates are estimated as 0.9, 1.5, and 1.7 nm per 1.0 × 10^13^ cm^−2^ of irradiation fluence for He^+^, Ne^+^, and Ga^+^ ions, respectively.

**Figure 3:**
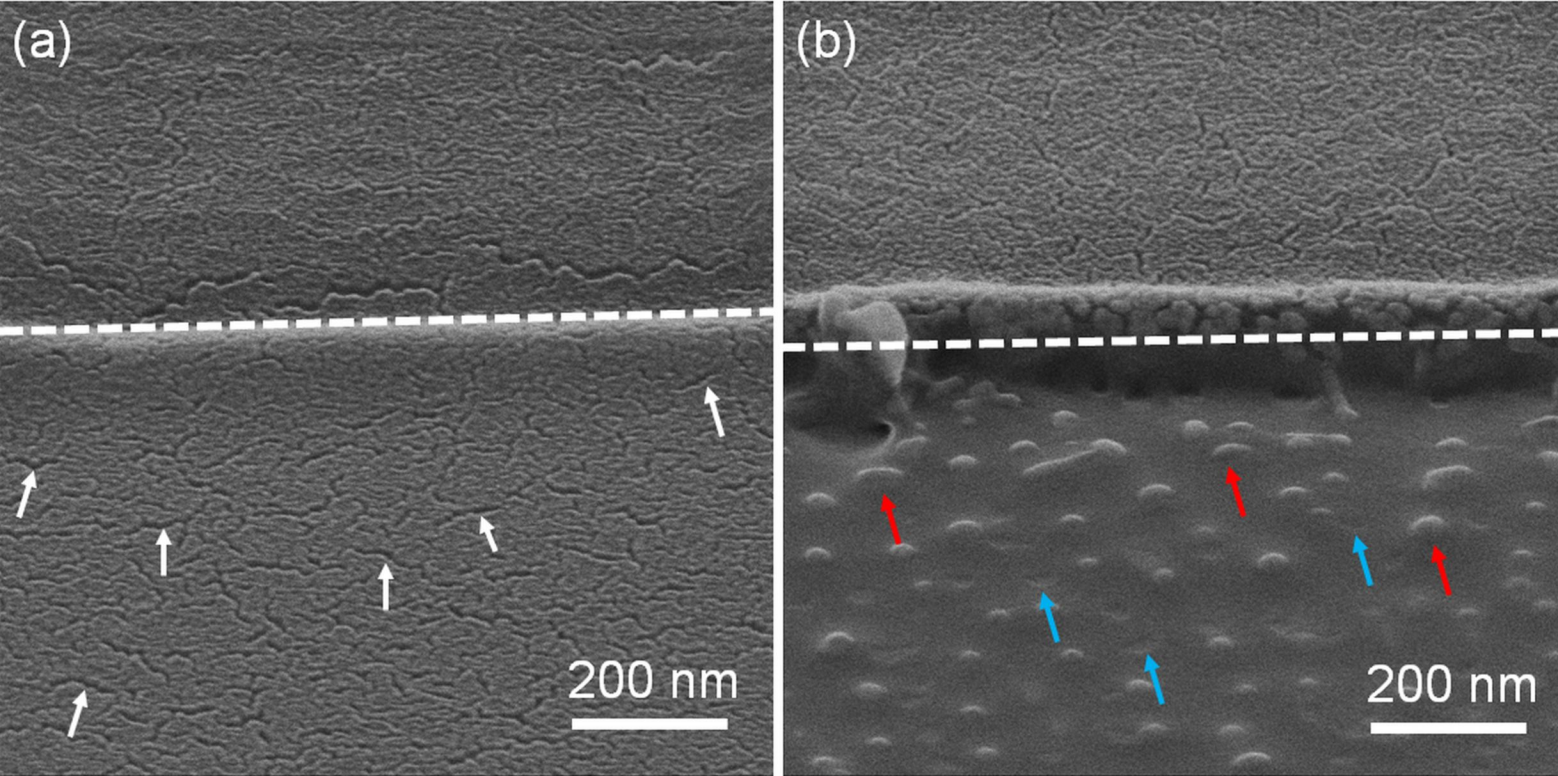
Helium ion microscopy (HIM) images of a 5 nm Pt_60_Pd_40_/200 nm PMMA sample irradiated at a fluence of 1.2 × 10^16^ cm^−2^ with He^+^ (a) and Ga^+^ FIB (b). In (a) and (b), dashed lines indicate the border between the irradiated (lower parts) and non-irradiated regions (upper parts). White arrows in (a) indicate some nanoscale cracks. In (b), red and blue arrows indicate local surface elevations and erosions, respectively. Both images are taken at a 54° tilt angle of the sample stage.

Figure 3 compares the surface morphology of 5 nm Pt_60_Pd_40_/200 nm PMMA samples in the case of a high-fluence irradiation with He^+^ and Ga^+^ ions. The metal film withstood the irradiation with He^+^ ions but it was removed by the irradiation with Ga^+^ ions. Besides, the Ga^+^-irradiated area is significanly rougher and characterized by erosions and spot-like elevations. For an irradiation fluence of 2.0 × 10^15^ cm^−2^, the values of the root-mean-square (RMS) roughness, measured with AFM in the irradiated areas, were approx. 0.7 and 4.4 nm for irradiation with He^+^ and Ga^+^ ions, respectively. The RMS roughness value of the pristine sample was approx. 0.6 nm. The irradiation with Ne^+^ ions also significantly roughens the surface and sputters away the metal film. The RMS roughness was approx. 3.1 nm after the irradiation with Ne^+^ FIB at a fluence of 2.0 × 10^15^ cm^−2^.

The sputtering efficiency of Ga^+^ and Ne^+^ ions is substantially higher than that of He^+^ ions [3] due to the significantly higher mass values of Ne^+^ and Ga^+^ ions (20 and 70 amu, respectively) when compared to He^+^ ions (4 amu). Thus, these results confirm that ions with intermediate and high mass values cannot be used in the scope of our nanopatterning technique since they destroy the pristine surface morphology and sputter away the pre-deposited films.

The observed increase in the surface descending rates and in the saturation level upon irradiation with Ne^+^ and Ga^+^ ions (Figure 2) indicate that, in those cases, both surface sputtering and subsurface volume shrinkage contribute to the changes in depth across the sample. According to our previous study [4], the reduction in surface height of the metal-coated PMMA surface is controlled by two major parameters. The first parameter is the irradiation fluence of He^+^ ions, which determines the total amount of radiation energy dissipated by the ions over their entire path in the sample. The second parameter is the thickness of the polymer layer, which determines the fraction of the total energy that is specifically dissipated in the polymer layer. An additional and important aspect is that polymer materials cannot shrink infinitely with an increase in the irradiation fluence and, at a certain fluence, the material capacity to shrink decreases, which explains the saturation effect in the case of irradiation with He^+^ ions. In the case of PMMA, the high irradiation fluence results in the formation of a compact carbon-rich material that can no longer shrink [13]. Taking this into consideration, we assume that a combination of several factors is responsible for the reduction in the surface height upon irradiation with Ne^+^ and Ga^+^ ions in comparison to the irradiation with He^+^ ions. The first factor is that heavier ions deposit a larger fraction of energy in the PMMA layer. This is depicted by the energy loss profiles simulated with the “Stopping and Range of Ions in Matter (SRIM)” software, as shown in Figure S1 (Supporting Information File 1). In the case of He^+^ ion irradiation, a significant fraction of the total ion energy is lost in the silicon substrate below the PMMA layer (Figure S1a and Figure S1b, Supporting Information File 1), meaning that this fraction is wasted with regard to defect generation inside the PMMA layer. In contrast, Ne^+^ and Ga^+^ ions lose their energy entirely in the PMMA layer; therefore, the total ion energy is utilized for generating the defects in this layer (Figure S1c–f, Supporting Information File 1). The second important factor includes the simultaneous contribution of ion sputtering and compacting processes near the surface. These events are significantly more pronounced when the irradiation is performed with ions with intermediate and high mass values, due to the higher density of the ion energy deposited near the surface in nuclear collisions.

The saturation effect in the case of irradiation with Ne^+^ and Ga^+^ ions shows that in the high-fluence range not only the material shrinking mechanism becomes inactive, but also the material sputtering becomes markedly slow. The estimated value of the surface depression rate in the saturation region of Ga^+^ ion irradiation is approx. 0.12 nm per 1.0 × 10^13^ cm^−2^ fluence. This value is approx. 14 times lower than the estimated surface depression rate for the irradiation with Ga^+^ ions in the low-fluence range. This value is also approx. eight times lower than the estimated surface depression rate in the low-fluence range of the irradiation with He^+^ ions in which only the shrinking mechanism occurs. These results are consistent with previously published results [13], which show that the formation of a highly carbonized layer on the PMMA surface significantly retards the sputtering process.

**Figure 4:**
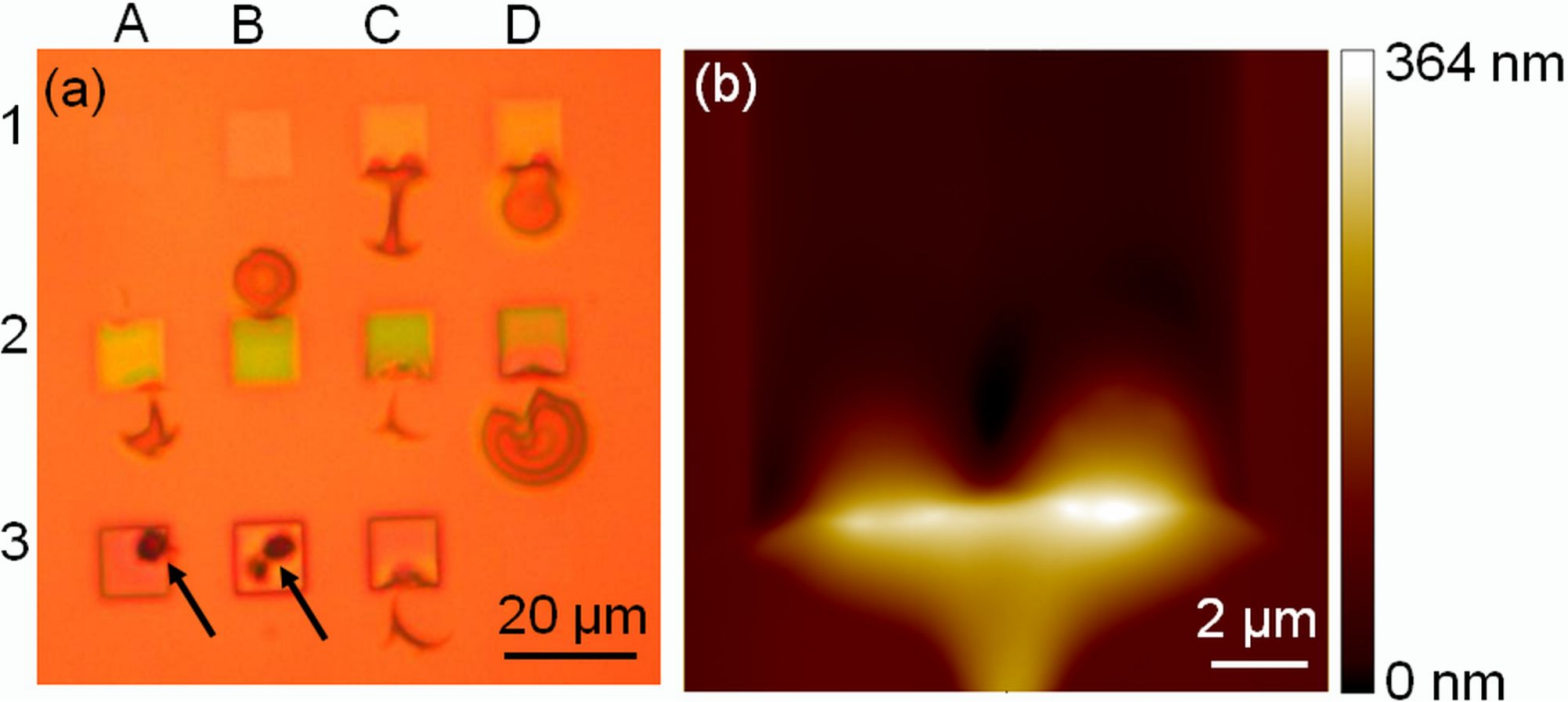
(a) Optical micrograph of an array of squares produced with He^+^/25 keV FIB in a 15 nm Au/200 nm PMMA sample. The irradiation fluence increases in the following raster scan order: A1 (3.3 × 10^13^ cm^−2^), B1 (2.0 × 10^14^ cm^−2^), C1 (3.7 × 10^14^ cm^−2^), D1 (5.3 × 10^14^ cm^−2^), A2 (1.0 × 10^15^ cm^−2^), B2 (2.0 × 10^15^ cm^−2^), C2 (2.5 × 10^15^ cm^−2^), D2 (5.1 × 10^15^ cm^−2^), A3 (7.6 × 10^15^ cm^−2^), B3 (1.0 × 10^16^ cm^−2^), C3 (1.3 × 10^16^ cm^−2^), and D3 (3.3 × 10^13^ cm^−2^). The lowest fluence (3.3 × 10^13^ cm^−2^) was irradiated in cells A1 and D3 to control the reproducibility of the obtained results. Black arrows indicate the holes in the Au film in the irradiated regions. (b) AFM image of the C1 square.

Figure 4 shows the results for PMMA samples coated with a 15 nm thick Au film and irradiated with 25 keV/He^+^ FIB. In contrast with previously published results for samples coated with 15 nm Pt_60_Pd_40_ films [4], the results shown in this work demonstrate that for samples coated with 5 nm Pt_60_Pd_40_ films, extensive delamination and bulging of the Au film from the substrate surface are observed in the irradiated cells and in the regions surrounding the cells. This is seen as changes in the color contrast of the cells in rows 1 and 2 in Figure 4a and confirmed by AFM imaging in Figure 4b. These effects are attributed to the accumulation of gases from radiolysis at the Au film/PMMA interface and to the pressure that becomes, at a certain fluence and at certain places, sufficiently high to delaminate and bulge the film. At higher fluence values (corresponding to the cells in row 3, Figure 4a), the bulges are almost inexistent, which can be explained by the appearance of holes in the irradiated regions (e.g., cells A3 and B3 in Figure 4a). This induces gas release and deflation of the bulges. These results demonstrate the importance of pathways for desorption of gases resulting from radiolysis. Moreover, our study shows that Au thin films (in our case, 15 nm thick) form very strong barriers for the permeation of gases and can withstand high degrees of stretching required for the observed bulging. Another remarkable result is the bulging outside the irradiated areas (Figure 4b), which is considered a result of bulge nucleation at the boundary between irradiated and non-irradiated regions, followed by an in-plane bulge propagation inside and outside the irradiated regions.

The results show that continuous films of low permeability cannot be patterned with the technique described here. In order to apply the technique, a gas-leakage path, for instance, in the form of an array of microholes, needs to be prefabricated in the films before irradiation. As an alternative to a continuous film, an array of discrete film features can also be pre-deposited onto the substrate.

### Irradiation of PC

**Figure 5:**
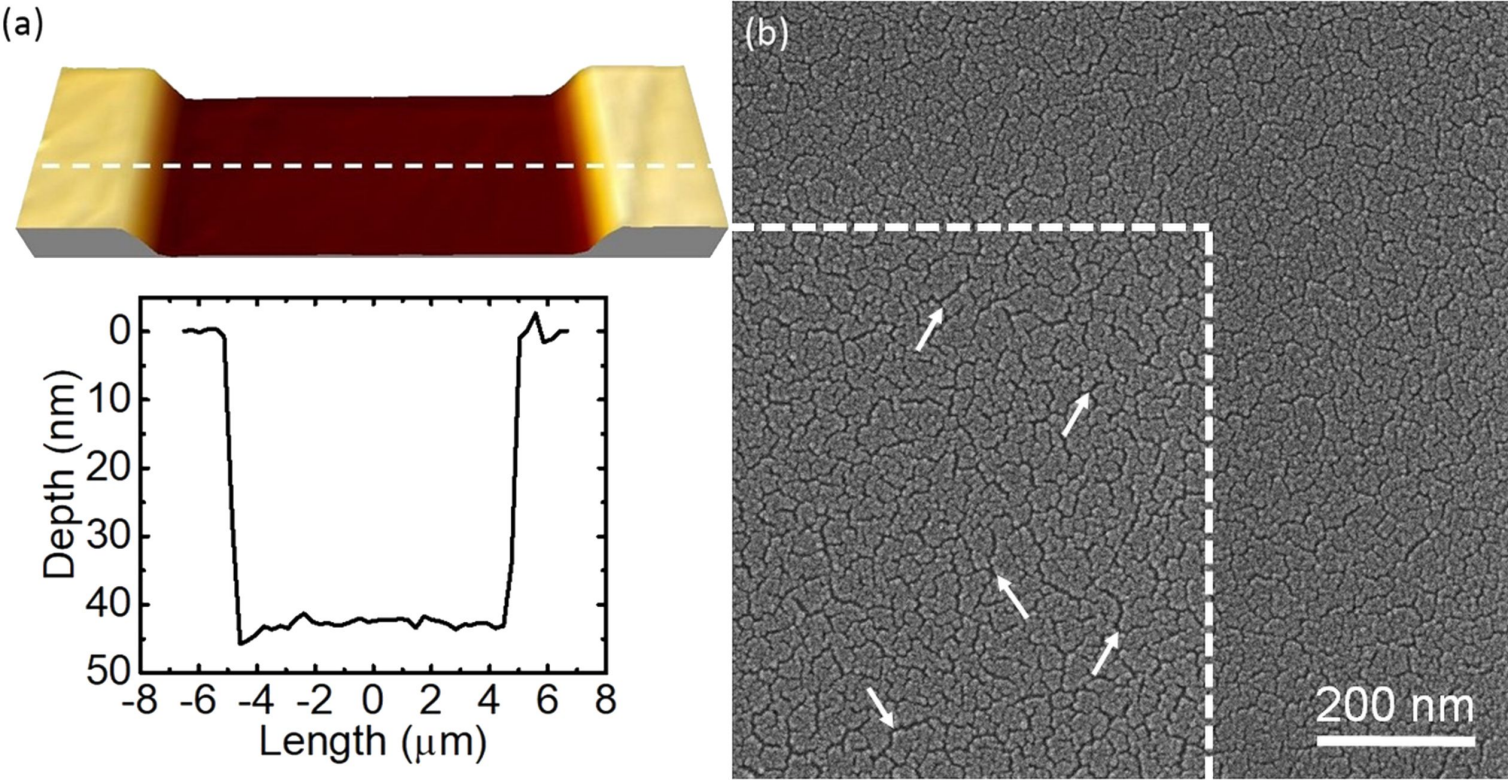
(a) AFM image and the corresponding depth profile of a part of the surface depression produced in a 15 nm Pt_60_Pd_40_/PC sample by irradiation with He^+^ FIB at an energy of 25 keV with a fluence of 2.0 × 10^15^ cm^−2^. The dashed line indicates the place of the depth profile. (b) HIM image of a part of the same depression, demonstrating the persistence of the metal film. White arrows indicate some nanoscale cracks in the Pt_60_Pd_40_ film in the irradiated region. Dashed lines indicate the border between irradiated and non-irradiated regions.

The results obtained for the irradiation with He^+^ FIB on 15 nm Pt_60_Pd_40_/PC samples appear to be similar to those obtained for the 15 nm Pt_60_Pd_40_/PMMA samples. AFM images and the corresponding depth profiles (Figure 5a) show that, within the entire fluence range, He^+^ FIB irradiation uniformly lowers the surface. High-magnification HIM images (Figure 5b) demonstrate the preservation of the metal film and the presence of cracks in the irradiated and non-irradiated areas of this film.

**Figure 6:**
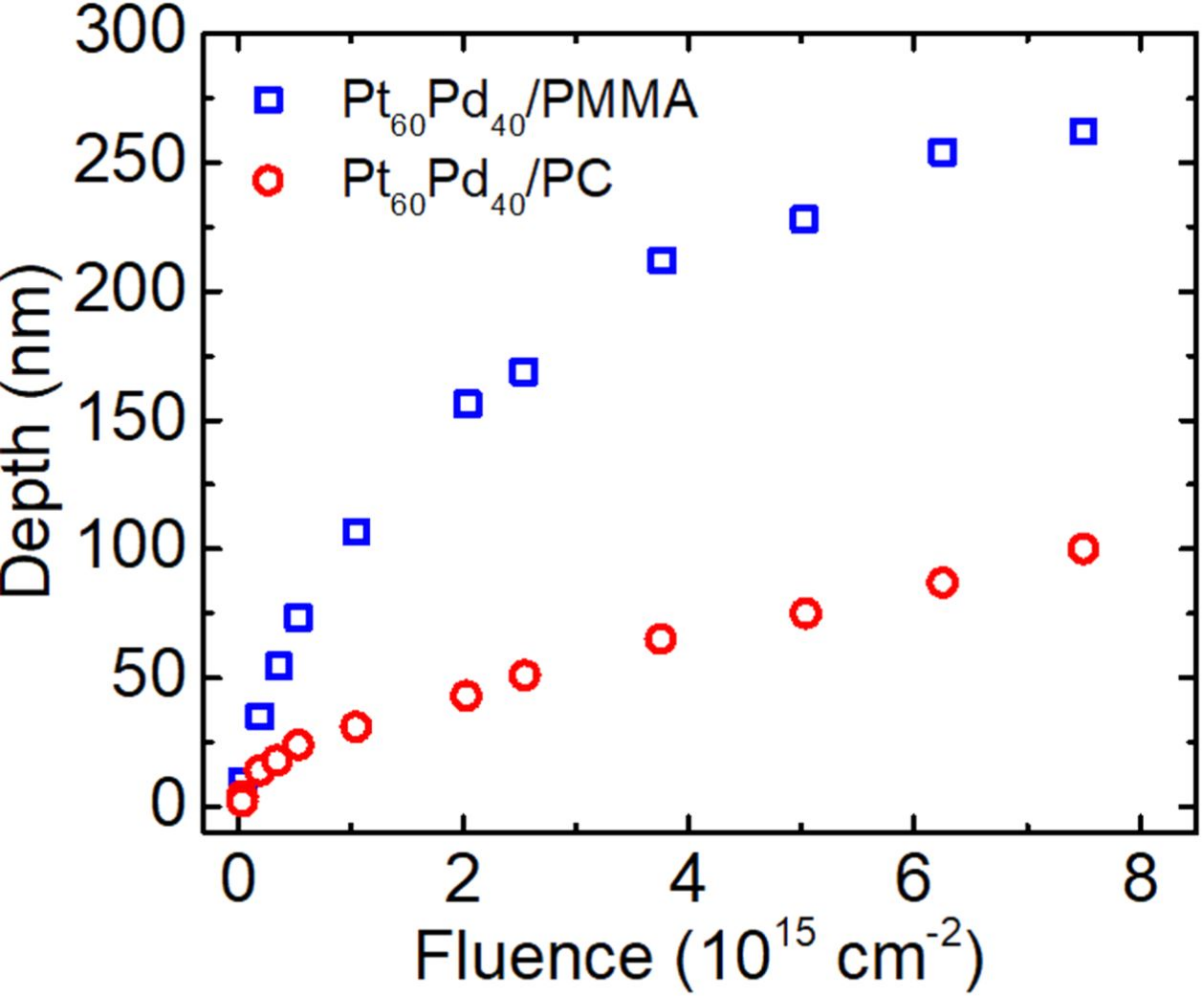
Fluence dependence of the irradiation-induced depth for the Pt_60_Pd_40_/PC sample (red circles). For comparison, the fluence dependence for a 15 nm Pt_60_Pd_40_/770 nm PMMA sample (blue squares) measured in our previous study [4] is also presented. The samples were irradiated with a 25 keV He^+^ FIB within a fluence range from 4.0 × 10^13^ to 7.5 × 10^15^ cm^−2^.

The dependence of the surface depression depth on the irradiation fluence for the 15 nm Pt_60_Pd_40_/PC sample is shown in Figure 6 (red circles) and compared to the fluence dependence for a 15 nm Pt_60_Pd_40_/770 nm PMMA sample (blue squares) obtained in our previous work [4]. In the latter, the 770 nm thick PMMA layer corresponds to a PMMA bulk substrate since the entire path of He^+^ ions is located within this layer (see Figure S2, in the supplementary material of [4]). The curves in Figure 6 have similar shapes. However, the depth-change rate and the absolute values as a function of the fluence are significantly lower in the case of PC substrates. As a result, the total depth change observed in the PC substrate at the highest dose (7.5 × 10^15^ cm^−2^) is approx. 2.5 times smaller than that in the case of the PMMA substrate. This difference can result from a combination of several factors related to the polymer structure and composition, as well from the structural response of these materials to irradiation. This requires a more extended study; however, within the scope of this article, we can conclude that the PC material is applicable for the suggested patterning scheme similarly to PMMA. Higher rates and values of the surface height reduction can be achieved by increasing the ion energy. These results are consistent with previous reports on chain scission, cross-linking, and material compacting under the exposure to different types of electromagnetic and corpuscular radiation [26–27].

### Irradiation of PDMS

**Figure 7:**
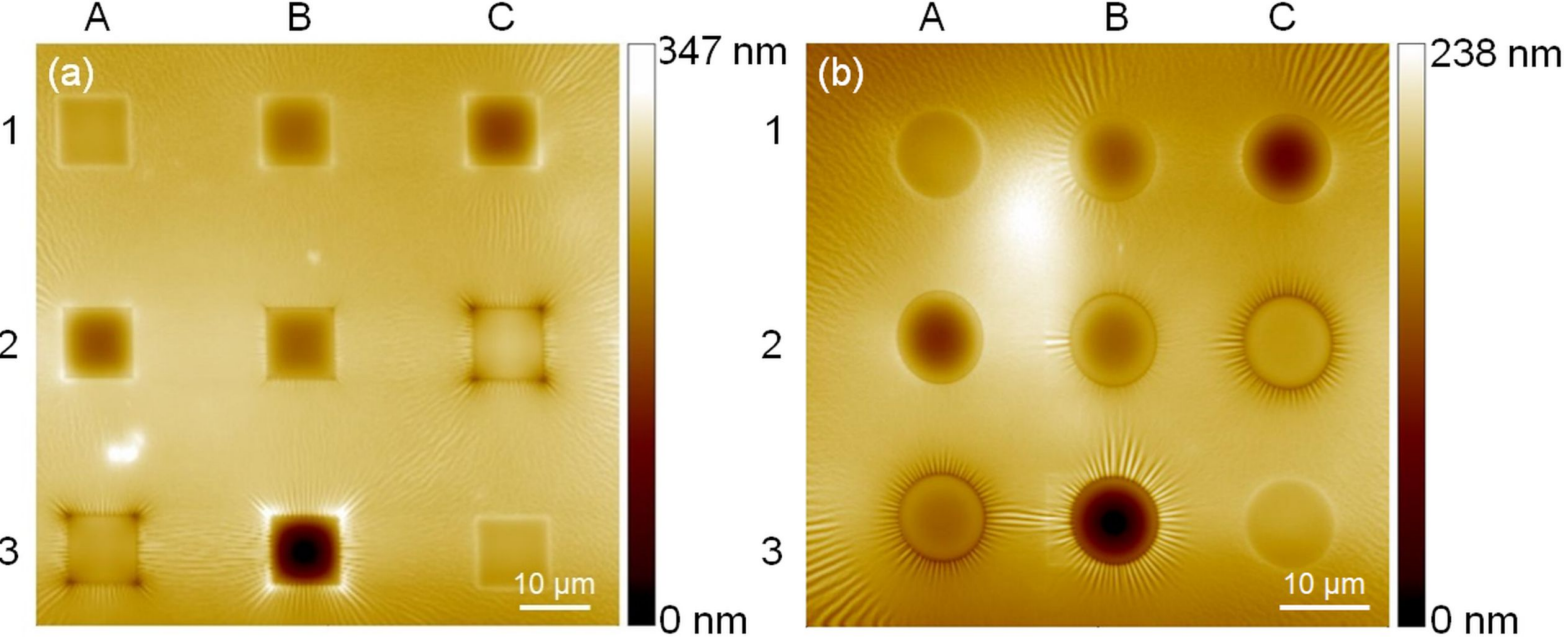
HIM images of square (a) and circular (b) arrays produced in 15 nm Pt_60_Pd_40_/PDMS samples by irradiation with different fluence values of 25 keV-He^+^ ions. In both images, the irradiation fluence increases in the following raster scan order: A1 (3.7 × 10^13^ cm^−2^), B1 (2.0 × 10^14^ cm^−2^), C1 (3.7 × 10^14^ cm^−2^), A2 (5.3 × 10^14^ cm^−2^), B2 (1.0 × 10^15^ cm^−2^), C2 (2.0 × 10^15^ cm^−2^), A3 (2.5 × 10^15^ cm^−2^), B3 (5.0 × 10^15^ cm^−2^), C3 (3.7 × 10^13^ cm^−2^). The lowest fluence was irradiated in two cells (A1 and C3) to control the reproducibility of the obtained results.

In terms of surface morphology and its dependence on the irradiation fluence, the results obtained for PDMS samples appear to be significantly different from the results obtained for PMMA and PC samples. Figure 7 shows examples of He^+^ ion irradiation of 15 nm Pt_60_Pd_40_/PDMS samples using square and circular patterns. Here, the irradiation of the PDMS samples with He^+^ FIB results in the formation of complex surface patterns. The patterns are composed of surface depressions in the irradiated areas and surface ripples surrounding the irradiated areas. The surface depressions have concave shapes, which are characterized by maximum surface depths at the geometrical centers of the irradiated squares (Figure 7a) and circles (Figure 7b). Some additional features (rectangular elevations at the left-hand sides of circles B2 and B3 in Figure 7b) are artifacts generated by scanning these areas with the He^+^ ion probe beam for imaging just after the irradiation.

**Figure 8:**
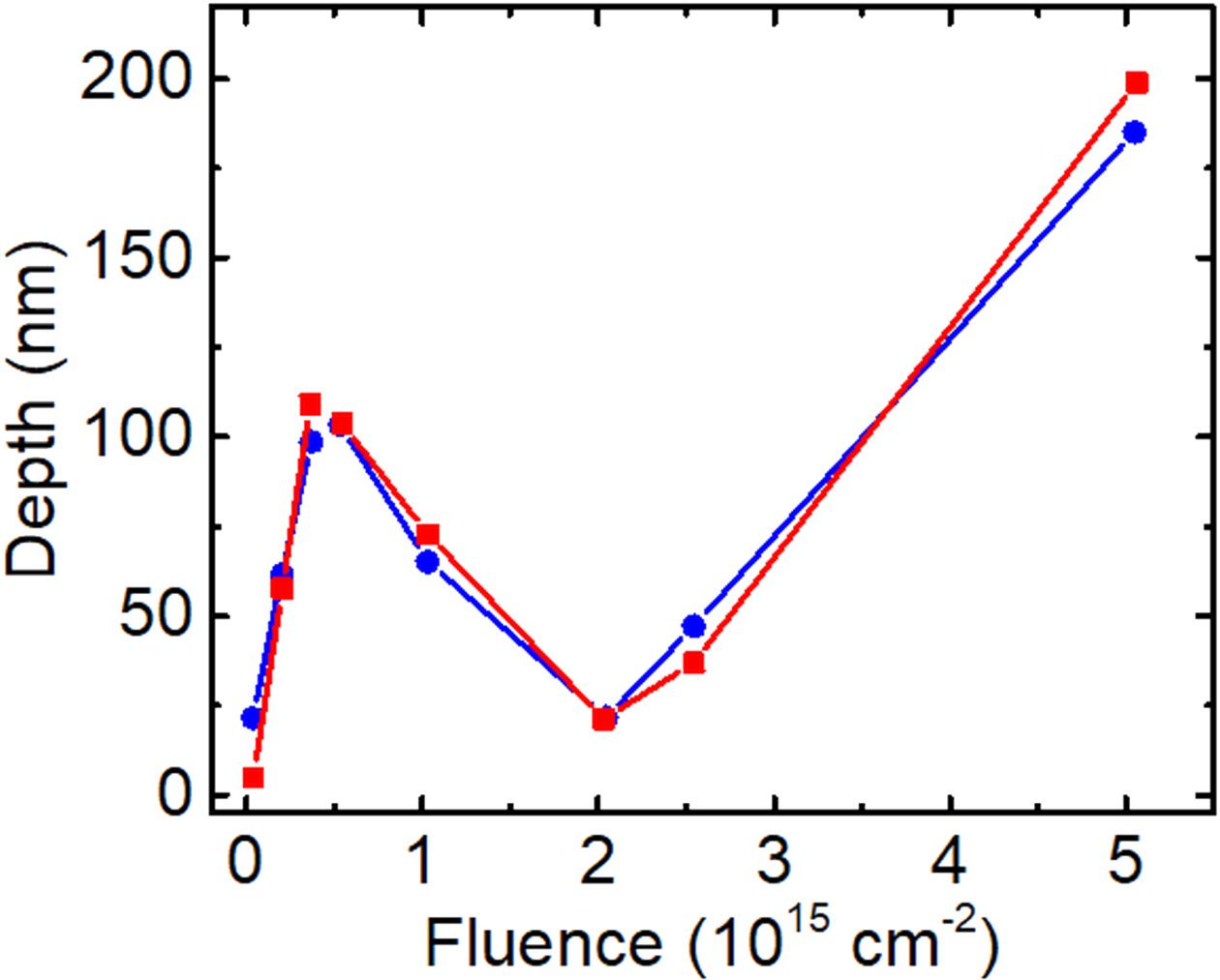
Fluence dependence of the irradiation-induced depth for a 15 nm Pt_60_Pd_40_/PDMS sample irradiated with a 25 keV He^+^ FIB within a fluence range from 3.7 × 10^13^ to 5.0 × 10^15^ cm^−2^. The depth is determined as the maximum depth within each irradiated area. Red squares and blue circles correspond to measurements performed in the irradiated areas of Figure 7a and Figure 7b, respectively.

The dependence of the maximum surface sinking depth on the ion fluence was measured in both arrays and is presented in Figure 8. In contrast to the fluence dependence for PMMA and PC samples, the graphs for PDMS samples include a region with a negative slope within an intermediate fluence range (from 3.7 × 10^14^ cm^−2^ to 2.0 × 10^15^ cm^−2^). This means that, at low- and high-fluence values the irradiated material volume pulls the surface down, whereas in the intermediate fluence range it pushes the surface back to the baseline position. In other words, with an increase in the irradiation dose, the PDMS material first shrinks, then swells, and then shrinks again.

The concave shapes of the surface inside of the irradiated PDMS regions can, to a large extent, be attributed to the elasticity of this material. A very low Young’s modulus for the Sylgard-184 PDMS material, ranging from 1.32 to 2.97 MPa [28], provides a long-range strain relaxation when compared to a short-range strain relaxation in non-elastic polymers, such as PMMA and PC. Therefore, instead of directly projecting the initially flat surface to another depth position, the irradiation-induced strain warps the pristine surface. The existence of long-range strain fields, sufficient for a significant deformation in the surface of our PDMS samples, is identified by the observation of ripple patterns around the irradiated areas. Other features associated with the strain fields in highly elastic materials are the sharp surface elevations or depressions at the corners of the irradiated squares in Figure 7b. Considering continuum mechanics, these features are places where mechanical stress can concentrate, resulting in the enhancement of local deformations.

In numerous previous studies, the occurrence of ripples (also referred to in the literature as ”wrinkles”) on the irradiated PDMS surface has also been reported. This is attributed to the formation of a silica-like, stiff skin layer that buckles to release the accumulated strain energy [29]. Remarkably, we did not observe any rippling in the irradiated areas within the entire fluence range. The only ripple patterns we observed were those generated by the stress field outside the irradiated regions, where there is no skin or any other structural or compositional material modification. One of the likely reasons for the absence of rippling inside of the irradiated areas in our PDMS samples is that the density of the total energy lost by He^+^ ions in the samples is not large enough to build up a sufficiently high stress to trigger rippling. This result is also interesting with regard to potential applications, because it opens up for a possibility of changing the surface curvature at the microscale while preserving the surface morphology at the nanoscale.

An even more remarkable result is the transition from shrinkage to expansion and then from expansion to shrinkage, as a function of the irradiation fluence (Figure 8). To explain this non-trivial surface kinetics, we assume that the structural transformations in the irradiated PDMS material depend on the mechanical strain induced in the irradiated polymer volume by the compacting process. In this case, the strain accumulates with the irradiation fluence and, at a certain fluence value, it reaches a threshold level above which changes in the structural reconstruction processes are observed. This leads to the transition from a compacting to an expanding phase. We emphasize that such a transition is favorable from a thermodynamic point of view because the volume expansion provides relaxation of the tensile strain specifically at the compacted regions. Therefore, it results in the reduction of the strain energy accumulated in the system. The energy minimization provides a thermodynamic force for the strain relaxation. In addition to this, the ion irradiation is needed to break atomic bonds and to lower the energy barrier for material expansion and relaxation. This entirely phenomenological model is consistent with the conclusions drawn in a previous study [25], in which swelling was observed in PDMS samples irradiated with a 2 MeV proton beam. In that the case, the irradiated surface was fabricated by cutting a piece of the PDMS polymer from a bulk sample. In contrast, the irradiation of a pristine PDMS surface of this sample resulted in material compacting. The authors explained that this difference was due to mechanical stress in the cut surface.

Other important aspects of the transformation of the PDMS sample induced by ion irradiation include irreversible changes in the material structure and in the elastic properties with an increase in the irradiation dose [21–25]. These factors can contribute significantly to set the threshold dose for the first strain-driven transition and can be responsible for the occurrence of the second transition followed by material shrinkage in the high-dose range. We also do not exclude that a certain accumulation of gases from radiolysis inside the irradiated volume occurs in our samples, and to some degree it can contribute to shape the dependence between depth and fluence presented in Figure 8. Further structural studies are required to complement the contribution of the radiation effects on the material parameters.

We emphasize that the suggested method for controlling the out-of-plane position of the surface features can be interesting for the fabrication of a range of microoptical and microfluidic devices and microelectromechanical systems (MEMS). Possible applications of the method for microoptical devices are discussed in detail in our previous work [4]. They include tuning the thickness of the dielectric layer in the metal–insulator–metal (MIM) structures used in linearly variable bandpass filters (LVBFs) [30–33]. The capabilities of PDMS substrates to induce multiscale surface curving are also very interesting regarding the manufacturing of photonic structures and microlens arrays. Nanometer-thick gaps and cavities with prefabricated nanostructures can be implemented in different schemes for nanoparticle control and separation in microfluidic systems [34], and as components of actuators or switches in MEMS [35–36].

Considering the future technological potential of the suggested method it is important to comprehend its limitations and advantages, especially in comparison to the direct 3D patterning with FIB milling. First of all, we noticed that the method is limited to the fabrication of low-aspect-ratio pattern features and does not impose a challenge to the area of high-aspect-ratio and high-lateral-precision 3D structures, in which FIB milling is a well-established technique for a broad range of materials. This limitation results from a combination of the limited capacity of the polymer substrates to shrink and a relatively large lateral straggle of He^+^ ions scattered in the bulk of the polymer materials. For instance, in the case of patterning with He^+^ ions (Figure 2), the maximum depth of a surface depression achieved in the high-fluence range is approx. 250 nm, while the projected lateral straggle of He^+^ ions is approx. 120 nm, as calculated with the SRIM code. The lateral straggle value gives a rough estimate of the smallest lateral size of the interaction volume involved in the shrinking process. Therefore, it estimates the smallest possible lateral size of the surface depression and the lateral precision achievable in this example. In addition, this method is only applicable to systems containing materials that shrink or swell under ion irradiation.

The most promising feature of the subsurface processing is its capability to pattern the out-of-plane position of objects (thin films and nanostructures [4]) prefabricated on the surface by other techniques, which can also include FIB milling. Thus, the subsurface processing can be exploited either as an editing tool or as one of the patterning steps in combination with different patterning processes for the fabrication of devices containing complex hierarchical structures composed of both nano- and micropatterns. Owing to the very small ion-induced sputtering impact, in the case of irradiation with He^+^ ions, the method is unsusceptible to a range of drawbacks associated with FIB milling, including redeposition of sputtered material, preferential milling, and edge effects [1,37]. Other harmful effects resulting from the interaction between ions and materials, such as structural damage and chemical modification that affect functional properties of the near-surface layers, are substantially reduced in comparison to FIB milling. Another advantage of the subsurface processing is that the local heating is negligible in comparison to ion milling with heavy ions [38–39]. This is due to the fact that the energy loss of He^+^ ions in the patterning approach is spread over larger stopping distances than that in the case of milling with heavy ions.

One of the major advantages of FIB milling with heavy ions, which is often cited in the literature, is its high patterning speed. However, the results of this work (Figure 2) show that in the case of milling PMMA polymers this advantage is no longer observed in comparison to the fluence rates achieved with He^+^ ions because of the substantial retardation of the milling process in the high-fluence range.

## Conclusion

In summary, the role of the subsurface and surface processes in the modification of the surface morphology of thin metal films was studied for three types of polymer substrates (PMMA, PC, and PDMS) by exposing these materials to He^+^, Ne^+^, and Ga^+^ FIBs in a Zeiss Orion NanoFab Helium Ion Microscope. We demonstrated the out-of-plane film patterning by the He^+^ FIB for all three polymer substrates coated with thin Pt_60_Pd_40_ films. The ion-induced material modifications in the bulk of the underlying polymer substrates generate film patterning while sustaining the essential film features. The irradiation of the Au-coated samples results in delamination of the Au thin film followed by its bulging and perforation, which points to the important role of available pathways for the desorption of gases resulting from radiolysis. The irradiation with Ne^+^ or Ga^+^ ion beams destroys the films and roughens the surface due to the prevalence of a sputtering process induced by ions with a high mass. In contrast to the flat surface depression observed in the case of PMMA and PC substrates, complex, multiscale surface patterns, and a transition from polymer compacting to polymer swelling were observed in Pt_60_Pd_40_-coated PDMS samples irradiated with He^+^ ions. The formation of complex surface shapes in this case is attributed to the inherent elasticity of the PDMS material. The transition from polymer compacting to polymer swelling is explained by the irradiation-induced mechanical strain accumulation followed by the relaxation of this strain at a certain critical value.

## Experimental

### Materials and samples

The PMMA and PDMS substrates used in this study were deposited onto the surface of blank silicon wafers. The deposition of PMMA was performed by spin coating in an RRT Lanz EBS 11 spin coater, in the same manner described in [4]. After the deposition, the samples were annealed at 200 °C for 90 s to remove solvent residuals. The PDMS polymer used was a two-component Dow Sylgard™184 silicone elastomer with a hardness value of 43 in the Durometer Shore scale. After mixing the components, the elastomer was deposited onto the surface of the silicon wafer, degassed in vacuum, and cured for 48 h at room temperature, which resulted in the formation of an approx. 0.8 mm thick PDMS layer. For the preparation of PDMS samples we intentionally avoided any spin coating, in order to fabricate a uniform layer that is free from any spinning-induced structural anisotropy [40]. The PC samples were 10 × 10 mm^2^ square pieces cut from 1.5 mm thick wafers of an optical-grade PC polymer manufactured by microfluidic ChipShop GmbH.

Thin metal films of either a Pt_60_Pd_40_ alloy or of Au were deposited onto the surface of the polymer substrates to study the patterning of these films by in-bulk processes. An important argument for using metal films is that these films prevent surface charging. The use of charge compensation by irradiation with electron beams can generate additional radiation damage in polymer materials. The Pt_60_Pd_40_ alloy films were deposited by DC sputtering as described in [4], in a Cressington 208HR sputter apparatus. The Au films were deposited with an e-beam in a Cryofox Explorer 600 physical vapor deposition system. We have been using very thin metal films (5 and 15 nm thick) to minimize the ion path length in these films and potential sputtering effects.

### FIB irradiation and sample characterization

The irradiation of the samples with He^+^, Ne^+^, and Ga^+^ ions was done in a Zeiss Orion NanoFab Helium Ion Microscope at a landing energy of 25 keV and with different fluence values ranging from 1.0 × 10^13^ cm^−2^ to 2.0 × 10^16^ cm^−2^. The beam current was kept at a value of approx. 1.7 pA for all irradiation experiments with He^+^ and Ne^+^ ions, and at approx. 2.0 pA for irradiation experiments with Ga^+^ ions. All irradiation experiments were performed in a single raster scanning mode with multiple passes and a beam dwell time of 2 µs. Arrays of 10 × 10 µm^2^ squares irradiated with different doses were used for measuring the dependence of the surface height on the irradiation dose, as previously described [4]. The distance between the square edges was kept at either 10 or 15 µm to avoid possible interactions between the irradiated areas, such as the overlaps originating from transverse ion straggle.

The samples were characterized with AFM and HIM. The measurements of the surface height were performed with a Veeco Dimension 3100 AFM instrument in the tapping mode. High-resolution imaging with a He^+^ ion-beam probe was performed using a very small beam current (below 0.1 pA) to minimize imaging artifacts from radiation damage generated by the probe beam.

## Supporting Information

SRIM simulations of collision and ionization in 5 nm Pt_60_Pd_40_/200 nm PMMA samples irradiated with He^+^, Ne^+^, and Ga^+^ FIBs.

File 1 SRIM simulations.
