# Supplementary material for: Out-of-plane surface patterning by subsurface processing of polymer substrates with focused ion beams
Source: Beilstein J Nanotechnol. 2020 Nov 6;11:1693–703. doi: 10.3762/bjnano.11.151 (PMC7653337; doi:10.3762/bjnano.11.151)
Supplement: File 1 — SRIM simulations. [file Beilstein_J_Nanotechnol-11-1693-s001.pdf]

BEILSTEIN JOURNAL OF NANOTECHNOLOGY

Supporting Information

for

Out-of-plane surface patterning by subsurface processing of polymer substrates with focused ion beams

Serguei Chiriaev, Luciana Tavares, Vadzim Adashkevich, Arkadiusz J. Goszczak and Horst-Günter Rubahn

Beilstein J. Nanotechnol. **2020,** *11,* 1693–1703. doi:10.3762/bjnano.11.151

SRIM simulations

License and Terms: This is a supporting information file under the terms of the Creative Commons Attribution License (https://creativecommons.org/licenses/by/4.0). Please note that the reuse, redistribution and reproduction in particular requires that the authors and source are credited.
The license is subject to the *Beilstein Journal of Nanotechnology* terms and conditions: (https://www.beilstein-journals.org/bjnano)

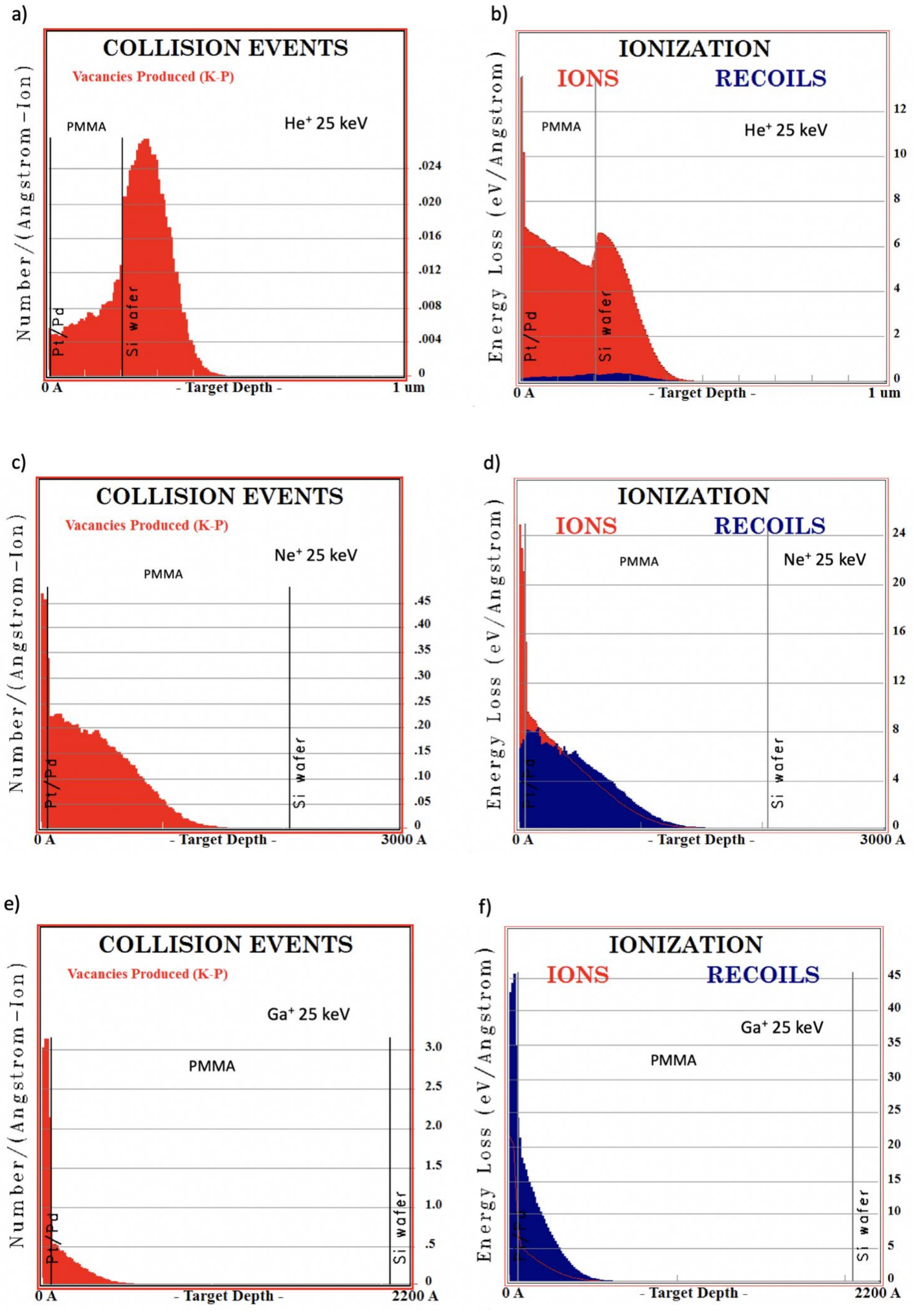

Figure S1: SRIM simulations of collision and ionization in the 5 nm $Pt_{60}Pd_{40}$/200 nm PMMA samples, irradiated with (a, b) He^+, (c, d) Ne^+, and (e, f) Ga^+ FIBs.
